# Fabrication and Mechanical Characterization of Hydrogel Infused Network Silk Scaffolds

**DOI:** 10.3390/ijms17101631

**Published:** 2016-09-26

**Authors:** Lakshminath Kundanati, Saket K. Singh, Biman B. Mandal, Tejas G. Murthy, Namrata Gundiah, Nicola M. Pugno

**Affiliations:** 1 Laboratory of Bio-Inspired & Graphene Nanomechanics, Department of Civil, Environmental and Mechanical Engineering, University of Trento, Via Mesiano 77, 38123 Trento, Italy; 2 Biomaterial and Tissue Engineering Laboratory, Department of Biosciences and Bioengineering, Indian Institute of Technology, Guwahati 781039, Assam, India; saket.singh@iitg.ernet.in (S.K.S.); biman.mandal@iitg.ernet.in (B.B.M.); 3 Departments of Civil Engineering, Indian Institute of Science, Bangalore 560012, Karnataka, India; tejas@civil.iisc.ernet.in; 4 Departments of Mechanical Engineering, Indian Institute of Science, Bangalore 560012, Karnataka, India; namrata@mecheng.iisc.ernet.in; 5 Center for Materials and Microsystems, Fondazione Bruno Kessler, Via Sommarive 18, Povo, I-38123 Trento, Italy; 6 School of Engineering and Materials Science, Queen Mary University of London, Mile End Road, London E1 4NS, UK

**Keywords:** silk scaffolds, tissue engineering, foam mechanics, permeability, mechanical infusion

## Abstract

Development and characterization of porous scaffolds for tissue engineering and regenerative medicine is of great importance. In recent times, silk scaffolds were developed and successfully tested in tissue engineering and drug release applications. We developed a novel composite scaffold by mechanical infusion of silk hydrogel matrix into a highly porous network silk scaffold. The mechanical behaviour of these scaffolds was thoroughly examined for their possible use in load bearing applications. Firstly, unconfined compression experiments show that the denser composite scaffolds displayed significant enhancement in the elastic modulus as compared to either of the components. This effect was examined and further explained with the help of foam mechanics principles. Secondly, results from confined compression experiments that resemble loading of cartilage in confinement, showed nonlinear material responses for all scaffolds. Finally, the confined creep experiments were performed to calculate the hydraulic permeability of the scaffolds using soil mechanics principles. Our results show that composite scaffolds with some modifications can be a potential candidate for use of cartilage like applications. We hope such approaches help in developing novel scaffolds for tissue engineering by providing an understanding of the mechanics and can further be used to develop graded scaffolds by targeted infusion in specific regions.

## 1. Introduction

Fibrous elastic proteins, like silk, have low density and combine the ability to undergo large deformation with high resilience which makes it an extremely attractive structural biomaterial for applications that combine high specific strength and toughness [1]. Silk, produced from cocoons of the silkworm *Bombyx mori*, is widely used commercially and in several biomedical applications due to its excellent mechanical properties and ease of availability. The versatility and ability of fabricating silk into complex three-dimensional shapes, material biocompatibility, and slow in vivo degradation rates together make it an attractive material for drug release applications and as tissue engineered scaffolds [2,3]. Such applications, however, require silk scaffolds with variable porosities and tunable mechanical properties which may influence their long term material viability [4,5,6]. 

Characterising the mechanical behaviour of such porous scaffold materials is crucial for their use in musculoskeletal applications that depend crucially on the strength, toughness, ability to undergo large deformations, and also support tissue growth [7]. In addition to these properties, scaffold porosity greatly influences interstitial liquid transport which is essential in sustaining cells within the tissue engineered constructs. Understanding the mechanical response of soft porous materials under a gamut of load conditions is an important mechanical consideration in scaffold design. Methods developed to understand the mechanics of fluid saturated biphasic materials were used to describe the behaviour of brain tissue [8], cartilage [9,10], and other hydrogels [11,12]. Ateshian and co-workers estimated the permeability of cartilage using a hyperelastic biphasic constitutive model with results from compression and creep tests [9]. Investigations into the load bearing capacity in combination with permeability are especially important in cartilage like applications. Cartilage not only distributes and transmits the load but also plays an important role in reducing friction by lubricating the interface [13]. According to the biphasic cartilage model, fluid film lubrication is facilitated by the drag forces of exuded interstitial fluid during static loading [14]. The surface lubrication improves with permeability in the superficial layer, resulting in low coefficient of friction [15]. Thus, permeability influences the lubrication at the interface and thereby influences the overall wear of the layer. More recently, compression experiments were used to quantify the creep behaviour of silk hydrogels based on the Terzaghi consolidation theory using mechanical testing over long time durations [16]. Thus, it is important to obtain optimum scaffold porosity, strength in combination permeability, by altering the concentration, density of cross-linking, and processing technique [17]. 

In this study, we fabricated individual network scaffold and filler matrix hydrogel using two different methods and combined them to obtain a composite scaffold by mechanical infusion. The mechanical properties of these scaffolds with variable microstructures were characterized using simple unconfined compression tests and later quantified according to their energy absorbing capabilities. We also predicted the mechanical behaviour of these silk scaffolds by modelling them as foams. Using confined creep compression experiments, we determined the scaffold consolidation behaviour by application of consolidation models over shorter times to examine the material’s creep behaviour and simultaneously determined their load dependent permeability. Our results showed that the composite scaffold has modulus similar to that of cartilage (0.3–0.5 MPa) [18]. Such studies help in designing and understanding the compression behaviour and failure mechanisms of tissue engineering scaffolds to achieve tailored microstructures, resulting in optimised mechanical multifunctional properties. 

## 2. Results

### 2.1. Unconfined Compression Experiments Show Differences in the Scaffold Properties

**Figure 1:**
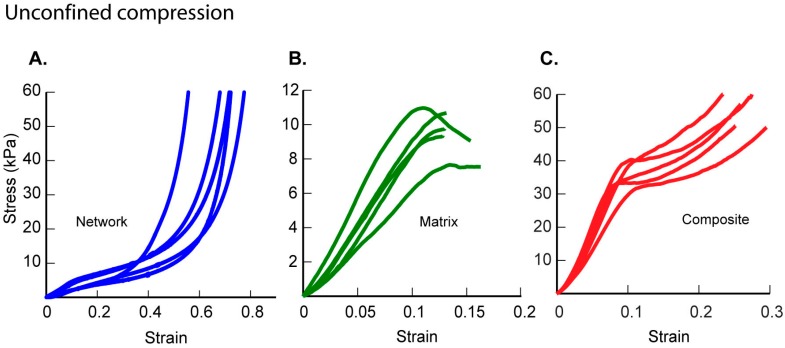
Results from unconfined compression tests of (**A**) network scaffolds (**B**) corresponding data from matrix hydrogels, and (**C**) composite scaffolds in the study (*n* = 5).

The stress-strain responses of porous network scaffolds were delineated into three regions: first, an initial linear elastic region characterized by elastic modulus. Second, a plateau region characterised by a plastic like strength; and finally, a stiffening response with the high values of stress increment for relatively small increases in strain (Figure 1A). In contrast, matrix hydrogels displayed a linear response and failed catastrophically at low strains (Figure 1B). The mechanical responses of composite scaffolds showed a plateau region which extended over a significantly smaller strain regime (Figure 1C). Yield stress, defined as the stress value in the stress-strain curve from which the stress decreases with increasing strains, was 9.6 ± 1.3 kPa for the filler specimens. Network scaffolds yielded at 3.8 ± 1.1 kPa without any catastrophic failure and exhibited a nonlinear stress-strain behaviour which was accompanied by significant stiffening after reaching 40% or higher strain. Yield stress (Table 1) for composite scaffolds was higher (41.1 ± 3.9 kPa) as compared to both the network and matrix materials (*p* < 0.05). Composite scaffolds showed a gradual increase in stress following the yield point unlike failure seen in the matrix. The elastic moduli, calculated using 3%–6% strain data, for the three scaffold groups clearly show significantly stiffer responses for the composite group as compared to the network and matrix groups. This is a noteworthy result showing that a composite scaffold material can achieve an elastic modulus higher than either of its components. Further, we used results from uniaxial unconfined stress-strain experiments to calculate the energy absorbed per unit volume of all the scaffold materials. The energy absorbed by the highly porous network scaffold was 3.94 ± 1.77 kJ/m^3^ and that of the matrix hydrogel was 0.46 ± 0.06 kJ/m^3^. The composite scaffold displayed better energy absorption ability with a value of 9.19 ± 1.3 kJ/m^3^.

### 2.2. Role of Interstitial Fluid in the Poro-Mechanics of Scaffolds

**Figure 2:**
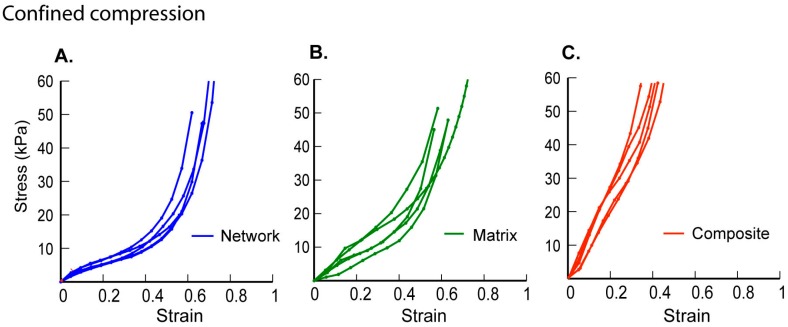
Confined compression results for (**A**) network scaffolds; (**B**) matrix hydrogels; and (**C**) composite scaffolds are shown to characterize the role of water in the overall mechanical response (*n* = 5).

Confined compression experiments were used to examine the role of fluid in the scaffolds. The mechanical response of network scaffolds was similar in both unconfined and confined tests, which may be related to the large elastic network structure coupled with high porosity (Figure 2A). In contrast, matrix hydrogels displayed mechanical behaviour characterized by a linear region followed by stiffening responses which were different from the unconfined compression results (Figure 2B). Composite scaffolds had mechanical behaviour characterized by a relatively longer linear region followed by a stiffening response (Figure 2C). Matrix hydrogels, which failed catastrophically under unconfined compression, did not fail even at ~80% strains under confinement. Similarly, composite scaffolds demonstrated a stiffer material response and could sustain strains in excess of ~40% at high loads. 

**Figure 3:**
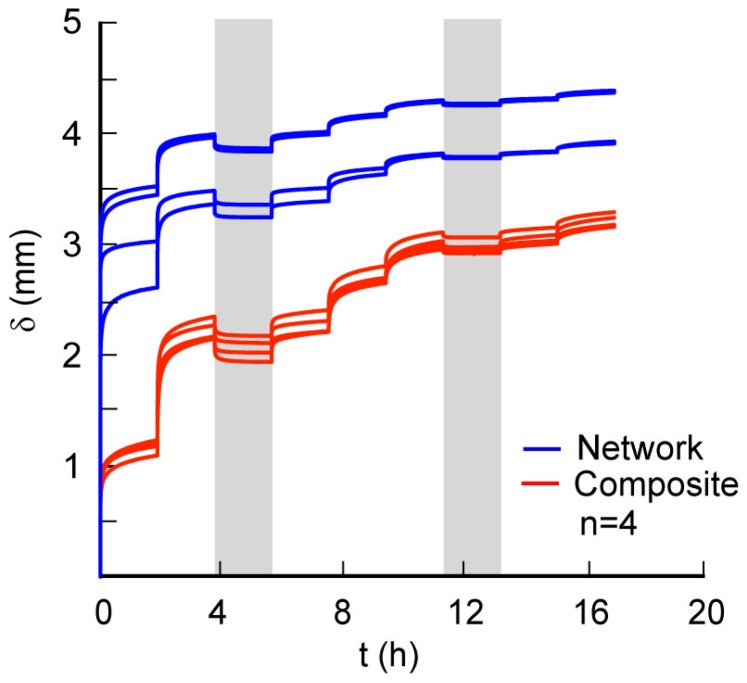
Changes in the specimen displacement during creep loading were obtained using an incremental loading sequence for network and composite scaffolds (grey columns highlighting the unloading steps).

**Figure 4:**
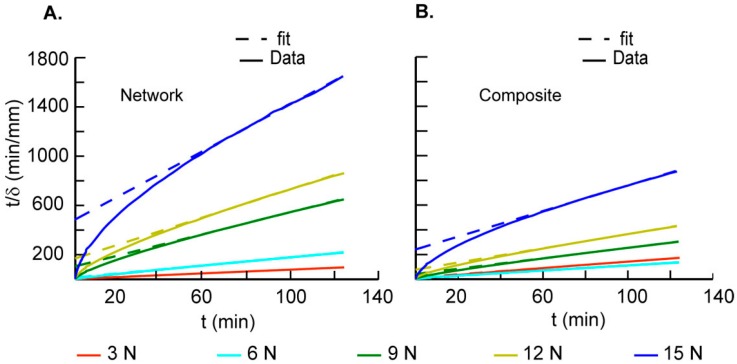
Data are shown for a representative (**A**) network and (**B**) composite specimen tested using confined creep loading as described earlier. Fits to rectangular hyperbola method (*r*^2^ > 0.98) were obtained and used to assess differences in the consolidation behaviours.

**Figure 5:**
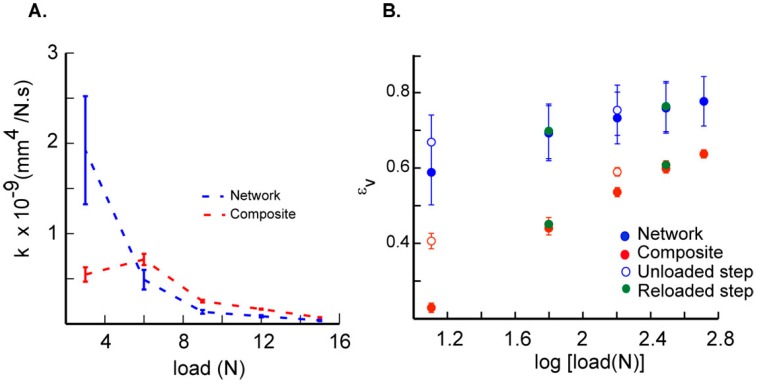
(**A**) Changes in the scaffold hydraulic permeability (*k*) with increase in load; (**B**) *ε*_v_ during loading are plotted to distinguish the rate of consolidation in network and composite scaffolds. Unloaded data points are also shown on the same figure.

Confined creep test results show variations in displacement with time for network and composite scaffolds that are characteristic of viscoelastic materials (Figure 3). Composite materials had more repeatable stress-strain responses as compared to network scaffolds which displayed variability in their responses. We suggest that this observed variability is due to variation in the interconnected porosity of the network scaffolds from sample to sample. In addition, the deformation of the scaffolds is not uniform throughout the height of the scaffold. Thus, variation in the distribution of the porosity in the height direction also contributes to higher initial compaction during the application on step load of 3 N, as seen in the creep deformation curves. These data also show a smaller change in displacements for each increase in step load as compared to the previous step; this change is related to the removal of fluid from the scaffold accompanied with scaffold densification. The converted creep data from each load step show the differences in network and composite scaffolds with their corresponding linear fits used to determine the parameters (Figure 4A,B). The average *C*_v_ values were highest for the network scaffolds at 3 N and decreased with increase in load (Table 2). Although composite scaffolds show a similar trend, we see a slight increase in *C*_v_ at 6 N (Table 2). In addition, the values for consolidation were higher for the composite as compared to the network scaffold at higher loads. Constrained moduli (*E*_c_) were calculated based on the plateau region obtained at each of the different loads [16]. Increased moduli at higher loads are linked to stiffening due to the loss of fluid during compression and a decrease in the material porosity due to collapse of the cell walls comprising the scaffold. Our results also show that the scaffold hydraulic permeability, *k*, was significantly higher for network scaffolds at 3 N as compared to composite scaffolds. However, these trends are reversed at higher loads which may be related to the higher densification in network scaffolds (Figure 5A). We next calculated the volumetric strain (*ε*_v_) in the composite and network scaffolds to assess variations in the material response (Figure 5B). These results show that *ε*_v_ was higher for network samples as compared to the composite scaffolds and increased with higher loads due to material densification. Further, the rate of change of *ε*_v_ was lower for network scaffolds because of the higher initial compaction at lower loads. The unloaded *ε*_v_ for each scaffold group show that the materials do not recover much but on reloading the strain was observed to increase slightly as shown in the same figure (Figure 5B).

### 2.3. Scaffold Microstructure and Porosity

**Figure 6:**
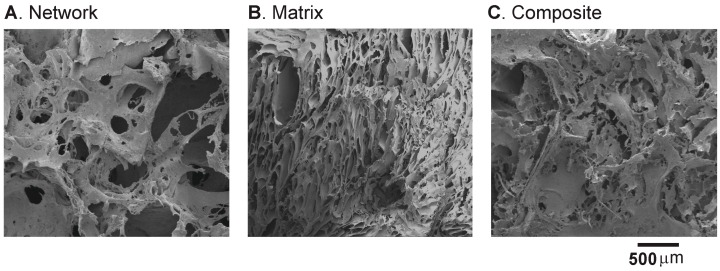
Scanning electron microscope (SEM) micrographs show microstructural differences. (**A**) Network scaffolds show highly porous structures with plate like cell walls; (**B**) Matrix hydrogels show relatively denser microstructures; (**C**) Composite scaffolds show dense areas of matrix hydrogel and the network cell walls.

**Figure 7:**
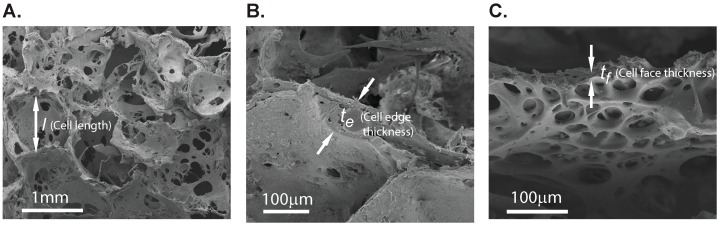
SEM micrographs of network scaffolds showing measurements of parameters used in modelling (**A**) basic unit cell length; (**B**) cell edge thickness, and (**C**) cell face thickness.

Scanning electron micrographs show visible differences in the scaffold microstructure. The network scaffolds had highly porous structures with the presence of plate-like cell walls (Figure 6A) whereas matrix specimens had denser microstructures with relatively much smaller pores (Figure 6B). In contrast, composite scaffolds were relatively homogenous with rougher surface domains and indistinguishable cell walls (Figure 6C). Measurements from the liquid displacement method show that porosities of network and composite scaffolds were 92.4% ± 0.8% and 67.2% ± 9.3%. The lower porosity in composite scaffolds was primarily due to the presence of matrix hydrogel infused into network scaffolds during fabrication. We measured the average cell dimension, cell edge thickness, and cell wall thickness to be 0.94 ± 0.25 mm, 123.25 ± 51.7 µm, and 13.69 ± 11.45 µm, using the scanning electron microscope (SEM) images, as shown in Figure 7.

## 3. Discussion

Developing tissue engineering scaffolds for cartilage like applications is a challenging task as it includes choice of a suitable material which has to be fabricated to achieve the desired microstructure, hydraulic permeability, and mechanical integrity. Earlier studies have reported fabrication of silk based network and hydrogel materials; combining the network response with filler hydrogel has not been examined to date. In this study, we fabricated a composite silk scaffold using a novel method that involved mechanical infusion of a hydrogel in a porous network scaffold. The resulting composite scaffold displayed enhanced mechanical properties indicating synergistic effects between the individual components. To explain and gain a better understanding of this unusual behaviour, we applied foam mechanics coupled with consolidation theory. 

### 3.1. Mechanics of Porous Fluid Filled Scaffolds 

Monotonic mechanical experiments are essential to characterize the material response and failure of the scaffolds. Our unconfined compression results show nonlinear stress-strain behaviours for network scaffolds in contrast to matrix and composite scaffolds which displayed relatively linear constitutive responses. Composite scaffolds yielded without sudden failure at strains of about 0.16 ± 0.01 as compared to matrix materials that failed catastrophically at strains of 0.12 ± 0.01. The addition of matrix hydrogels to network scaffolds hence presents several advantages via the composite ability to withstand high loads at small strains without visible damage in their microstructures. Our results also showed that the composite scaffold has significantly increased energy absorbing capabilities as compared to both of its constituents. The composite elastic modulus was higher than the values measured in human cartilage [19]. Thus, the composite scaffolds can be designed to match the mechanical properties of desired human cartilage properties by altering composition and fabrication parameters. 

Our results from unconfined compression displayed an unusual behaviour of the composite scaffold with significantly higher elastic modulus and yield strength as compared to both of its components. Using the simple rule of mixtures, we have estimated the contribution from the porous network scaffold material and found that it behaves more like fibre reinforcement with modulus and yield strength of about 4.48 MPa and 0.42 MPa, respectively. The elastic modulus value was close to that of a silk thin film [20], with a thickness in the order of the network scaffold wall thickness, as estimated from SEM images. Such results were obtained in silk fibre reinforced silk hydrogels fabricated with the goal of improving the hydrogel mechanical properties. The results suggested that there is a critical fibre length of ~500 µm that resulted in the efficient load transfer to obtain a composite with enhanced mechanical properties [21]. Also, the study suggests that using the same material for both the fibre and the hydrogel results in better mechanical properties because of better adherence of fibres due to improved molecular interactions. We conjecture that similar interactions would enhance mechanical properties of the composite scaffolds in the study. We then examined the applicability of foam mechanics to understand the deformation mechanism of these scaffolds. The predicted network elastic modulus was 25.3 kPa, which is close to the experimentally measured modulus (27.2 kPa, see Table 1). We further proceeded with the prediction of the composite elastic modulus using the concept of filled closed foam. In this model, the elastic modulus of the filled foam is estimated by adding contributions from cell edge bending and cell face stretching due to filled material expansion during compression, and the resistance from the filler material. Substitution of the known values in Equation (4) showed that the elastic modulus is a function of *ϕ* (Equation (5)), which is the volume fraction of the cell edges. We have estimated this fraction to be in the range of 0.182 ≤ *ϕ* ≤ 0.962 by substituting the values of thickness and size of the unit cell measured from the SEM images in Equation (6). The composite elastic modulus was determined to be in the range of 439.5 to 476.0 kPa, values which are similar to those obtained experimentally. Similar behaviour was observed in the deformation of 3D printed microfiber reinforced hydrogels that resulted in the improvement of stiffness by five orders of magnitude [22]. The resulting synergistic effect was an outcome of the fibres resistance to elongation in tension during the expansion of the filled hydrogel in compression. 

### 3.2. Consolidation and Permeability of Silk Scaffolds

Consolidation and hydraulic permeability are essential parameters in musculoskeletal tissue engineering applications such as cartilage and nucleus pulposus. Earlier studies have characterized the mechanics of fluid saturated porous materials using consolidation theories developed for soils saturated with fluid under the application of load [23]. Removal of water from the porous network, accompanied by active adaptation of the skeletal matrix to applied loads, yields the densification response of the structure which is characterized using a consolidation ratio. Kluge and co-workers performed creep experiments on silk hydrogels and modelled the consolidation response as diffusion, following an approach proposed by Terzaghi. They used the Casagrande method to estimate the rate of consolidation of fluid filled porous solids [16]. Because of difficulties in identifying the end of primary consolidation, which is required in the Casagrande method, we explored the applicability of the rectangular hyperbola method [24] to model the creep results in our study.

Table 2 shows *C*_v_ values calculated at each load point that show a steady decrease from 3 N to 15 N for the scaffolds. Higher values of *C*_v_ at 3 N for the matrix scaffolds (0.88 ± 0.28 mm^2^/min) are due to their high porosity as compared to denser composite scaffolds. At each other applied load, the *C*_v_ for network scaffolds was lower as compared to composite samples and is related to the higher densification of porous network scaffolds. In contrast, composite scaffolds show a slight increase in *C*_v_ at 6 N (0.88 ± 0.04 mm^2^/min) as compared to that at 3 N (0.65 ± 0.03 mm^2^/min). Similarly, results show that the measured hydraulic permeability was significantly higher for network scaffolds as compared to composite scaffolds in the first load step whereas trends are reversed at higher loads (Figure 7B). The relatively high hydraulic permeability may be related to the unbound water in the network scaffolds [25]. The measured hydraulic permeability values of the composite scaffold were three orders of magnitude higher than that of human cartilage measured at an order of magnitude higher load [18]. Combining network scaffold with the matrix material, however, presents advantages in controlling the hydraulic permeability and in altering the energy absorbing characteristics of composite scaffolds to permit a higher load bearing capability. The application of findings from this study and the novel fabrication technique may help in designing scaffolds that match the cartilage individual layers mechanical response in combination with desired permeability.

## 4. Materials and Methods 

### 4.1. Preparation of Aqueous Silk Solution

Fresh *Bombyx mori* silkworm cocoons were collected from the Khanapara sericulture farm in Guwahati, Assam, India, cut into small pieces, degummed for 20 min in sodium carbonate solution (Sigma-Aldrich, St. Louis, MO, USA), and rinsed in MiliQ water (Merk, Mumbai, India). Treated material was dried at room temperature followed by an additional treatment with 9.3 M LiBr (Sigma-Aldrich) solution for 4 h at 60 °C. The resulting solution was dialyzed against MiliQ water using 12 kDa membranes and the dialysate was centrifuged at 10,000 rpm for 5 min. The final concentration of silk solution was determined by gravimetric method and used in scaffold fabrication. The concentration (% *w*/*v*) is defined as the percentage of weight of the solute (g) divided by the volume of solution (mL).

### 4.2. Fabrication of Scaffolds

**Figure 8:**
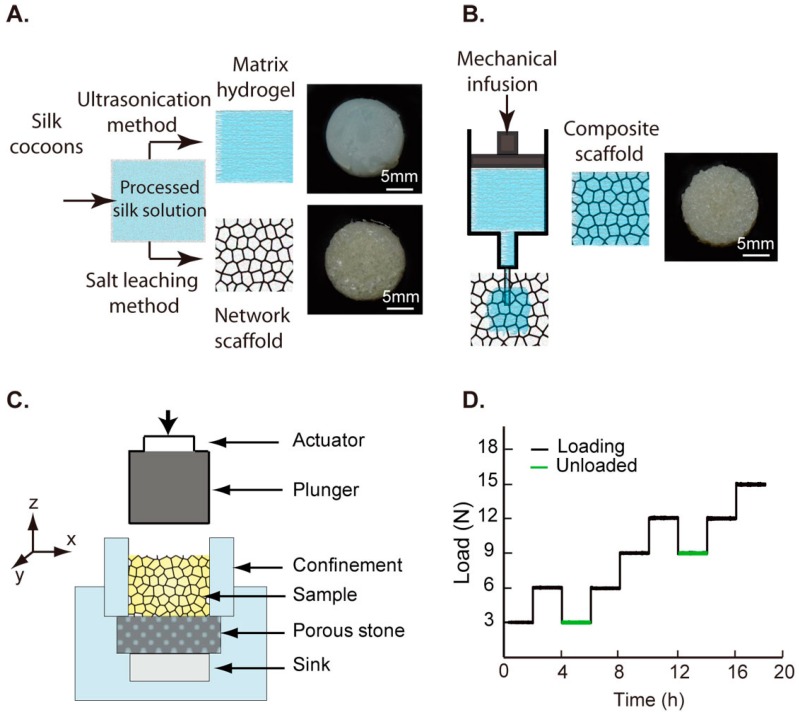
(**A**) Fabrication routes used for preparation of matrix and network samples, with a representative sample to highlight morphological differences; (**B**) Schematic of the mechanical infusion technique used for fabrication of composite scaffold, with a representative sample; (**C**) Custom confined compression chamber with porous stone was used to constrain the specimen for the mechanical experiments; (**D**) An incremental load sequence was performed in the confined compression chamber and includes two unloaded profiles to quantify scaffold recovery.

Three different scaffolds were prepared in this study. First, aqueous extracted silk fibroin was used to fabricate a pure 8% *w*/*v* scaffold, called “network”, using a salt leaching method (Figure 8A) [26]. Briefly, granular NaCl was added to 2 mL of 8% *w*/*v* silk solution in circular Teflon^®^ moulds (ThermoFisher Scientific, Waltham, MA, USA). Specimens were dried at room temperature overnight, followed by 12 h ethanol treatment. Silk networks were soaked in MilliQ water to remove salt and obtain a porous 3D scaffold. Second, “matrix” hydrogels were prepared by pouring 2 mL sonicated 4% *w*/*v* silk solutions into Teflon^®^ moulds (Figure 8A). Finally, “composite” scaffolds were fabricated by injecting 2 mL sonicated 4% *w*/*v* silk hydrogel matrix into porous network scaffolds (Figure 8B). Specimens were stored in 70% ethanol until subsequent use.

### 4.3. Measurement of Specimen Porosity and Scanning Electron Microscopy

The specimen porosity was measured using the liquid displacement method. In this, we measured the initial volume of the liquid (*v*_1_), liquid level rise following 5 min immersion of sample in ethanol (*v*_2_), and the drop in liquid level after specimen removal (*v*_3_) using a camera (Nikon D7000, Bangkok, Thailand). Porosity, P, defined as the ratio of volume of open pores in the scaffold to the entire volume, is given as:

(1) $$P=\frac{v_{3}-v_{1}}{v_{3}-v_{2}}$$

Scaffold density was obtained by measuring the specimen weight and dimensions. To visualize the microstructure, scaffold samples were sectioned using a scalpel, lyophilized, mounted on an aluminium stub using double sided carbon tape (Electron Microscopy Sciences, Hatfield, PA, USA), and placed in a desiccator to avoid moisture absorption. Specimens were transferred to a sputter coater (Bal-Tec SCD 500, Balzers, Liechtenstein), purged thrice with argon, and coated with gold. A scanning electron microscope (Quanta 200 ESEM, FEI, Eindhoven, The Netherlands), with accelerating voltages from 5 to 10 kV, was used to image the specimens.

### 4.4. Mechanical Testing 

#### 4.4.1. Unconfined and Confined Compression Experiments to Characterize Stress–Strain Properties 

Ethanol stored samples were rehydrated for 2 h in distilled water at room temperature. Mechanical tests were performed using a Bose Electroforce^®^ 3200 (Bose Corp., Eden Prairie, MN, USA) instrument equipped with two parallel compression platens. Displacements were measured using linear variable differential transformer (LVDT) and the forces from a load transducer (Bose Corp., ±22.5 N). Specimens for monotonic compression experiments were preloaded (0.3 N) to ensure proper seating of the platen on the specimen surface and the scaffolds were preconditioned using thirty cycles of 10% strain at 0.05 Hz. Unconfined compression tests were performed on five samples from each category at 0.1 mm/s until either the sample failed or the load limit of the transducer was reached. Engineering stresses (σ) were calculated as the ratio of the applied load to unloaded cross sectional area. Axial strains (*ε*) were defined as the ratio of elastic displacement to the initial specimen height. The stress-strain data were used to compute resilience, as energy absorbed per unit volume (*W*) by the scaffolds which can be used to assess their shock absorbing capabilities. Thus,

(2) $$W=\int_{0}^{\varepsilon_{m}}\sigma d\varepsilon$$

where *ε*_m_ is failure strain. In the case of the network scaffolds, the failure is defined as the strain value from which the densification of the porous network is identified using the graph [27]. Confined compression tests were performed on five samples from each category using a custom-built acrylic cylindrical confinement. The specimen was placed on a porous stone to permit excess water drainage and a stainless steel plunger was used to apply loads on the specimen (Figure 8C). Preliminary experiments were performed at different displacement rates to minimize the effects of fluid back pressure during loading. Data are reported from tests which were performed at 0.01 mm/s and room temperature.

#### 4.4.2. Confined Creep Experiments to Assess Material Permeability 

Samples (*n* = 4) from each category were preloaded (0.1 N) to ensure proper seating of the platen and loaded at a rate of 3 N/s. Confined creep tests were performed using step loads of 3 N increments, including a 125 mins hold time at each step, until a maximum load of 15 N (Figure 8D). All tests were performed at room temperature. Incremental step loads were selected to explore time dependent responses over the different deformation regimes and assess applicability of consolidation models in understanding the mechanics of fluid-filled scaffolds. Actuator displacements were used to obtain changes in sample heights during loading. In addition to monotonic creep loading, two load points were used to characterize scaffold recovery by unloading from a set load to the previous step.

#### 4.4.3. Statistical Analysis

We performed statistical analyses to test for differences in the mechanical properties between different groups using ANOVA with Bonferroni comparison using the *multcompare* function in MATLAB. A *p* value of <0.05 was considered significant in reporting these results.

## 5. Modelling

### 5.1. Foam Mechanics

**Figure 9:**
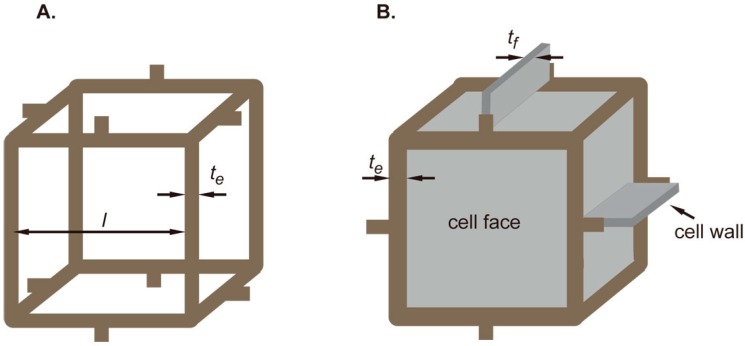
(**A**) A representative basic cubic unit cell used for predicting the modulus of network scaffold; (**B**) A corresponding closed-foam unit cell showing the cell edges and cell face thickness used for predicting the modulus of the composite scaffold.

The mechanical behaviour of the scaffolds was predicted by modelling them as foams with a simple cubic unit cell of unit length, *l*, as a building block (Figure 9A). The elastic modulus of the porous network scaffold is estimated using the following equation [28]:

(3) $$\frac{E^{*}}{E_{s}}={\left(\frac{\rho^{*}}{\rho_{s}}\right)}^{2}$$

where $E^{*},\;\rho^{*},\;E_{s},\;\rho_{s}$ are elastic modulus and density of the network scaffold and of the composing solid, respectively [28]. The density value of silk was taken as 1.3 g/cm^3^, as reported in the literature [29]. The elastic modulus of the composite $\left({E_{c}}^{*}\right)$ was estimated assuming it to be a filled closed-cell foam (Figure 9B) using the following equation [30]:

(4) $$\frac{{E_{c}}^{*}}{E_{s}}\approx \left\{\phi^{2}{\left(\frac{\rho^{*}}{\rho_{s}}\right)}^{2}+\left(1-\phi \right)\left(\frac{\rho^{*}}{\rho_{s}}\right)\right\}+\frac{{E_{m}}^{*}}{E_{s}}$$

where, *ϕ* is the solid fraction of material in the cell edges and ${E_{m}}^{*}$ is the modulus of the hydrogel matrix. In the right hand side of the equation, the first term represents the contribution from cell edge bending and the second term represents the contribution from cell face stretching. The third term is the additional contribution coming from the filled hydrogel matrix, assumed to behave like a liquid. Using the values of densities and elastic modulus of the solid and network materials, Equation (4) is simplified as:

(5) $${E_{c}}^{*}=0.0259\phi^{2}-0.076\phi +0.481\;[\mathrm{MPa}]$$

The solid fraction (ϕ) of the edges can be estimated using the following equation [30]:

(6) $$\phi =\frac{t_{e}^{2}}{t_{e}^{2}+\frac{n_{f}}{n_{e}}t_{f}l}$$

where $t_{e}$ and $t_{f}$ are thickness of the edge and face of the unit cell, respectively, as shown in Figure 9B. l is the length of the cubic unit cell side. $n_{f}$ is the number of faces that meet at an edge and $n_{e}$ is the number of edges per face in a unit cell.

### 5.2. Mechanics of Fluid Filled Materials

**Figure 10:**
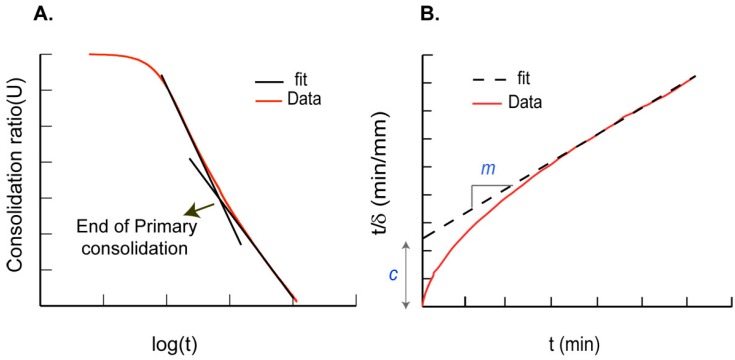
(**A**) Region for assessing transition from primary to secondary consolidation is shown in a schematic using the Casagrande method; and (**B**) a schematic showing the linear fit to the data using the rectangular hyperbola method.

The deformation response of a biphasic material under constant load depends on the individual stress-strain response of the structural phase in addition to the rate of fluid removal under loading. Reduction in volume experienced by a biphasic material with application of stress over time is defined as consolidation. In a one-dimensional consolidation setup, the ratio of changes in specimen height to its initial height is referred to as the consolidation ratio (U), which is used to describe the compression response. In a standard consolidation process, we observe three primary regions under mechanical loading of biphasic materials, which correspond to the pressure in the fluid trapped inside the pores of the solid. First, an initial short compression region wherein the entire applied load is carried by the fluid component, followed by a linear primary compression region that is characterized by the transfer of load from the fluid to the solid component of the scaffold. Finally, there is the secondary compression plateau region, when the entire load is supported by the structural solid scaffold alone. The point of transition from primary to secondary compression under the loss of varying pore fluid pressure (*p*(*z,t*)) is identified using a parameter termed as the consolidation coefficient (*C*_v_), which is given by:

(7) $$C_{v}\frac{\partial^{2}p}{\partial z^{2}}=\frac{\partial p}{\partial t}$$

where, *z* is a location in the sample at time *t*. Data from uniaxial confined creep experiments were used to obtain the consolidation response of fluid saturated materials. The rigid specimen confinement in these experiments permits deformation in the longitudinal (*z*) direction alone which is required in the quantification of density or volume changes, which may thus be directly linked to changes in displacement [31]. Traditionally, *C*_v_ is calculated by the application of the Casagrande method followed by the application of Terzaghi theory [31]. The Casagrande method is used to determine the end of primary consolidation by identifying changes in the slope of the consolidation ratio with time (Figure 10A). This method typically relies on experimental data obtained from specimen loading over long durations which may lead to specimen dehydration. In contrast, the alternate rectangular hyperbola method presents a distinct advantage in using relatively short duration creep loading results to determine *C*_v_ [24]. We tested the applicability of both these methods to model the consolidation behaviours of the silk scaffolds in this study.

In the rectangular hyperbola method, creep data from each load step was converted into the inverse of velocity (ratio of displacement with time) as a function of time to compare differences in material densification and compute the consolidation coefficient (*C*_v_). A straight line was fit to the end linear region of this curve to determine the best fit parameters of the slope (*m*) and the intercept (*c*) for each creep response (Figure 10B). The consolidation coefficient (*C*_v_) was calculated using the determined parameters and the initial sample height (*H*) using the following equation [24]:

(8) $$C_{v}=0.24\;mH^{2}c$$

The material hydraulic permeability (*k*) is estimated using the consolidation response during incremental scaffold loading and the constrained modulus (*E*_c_) calculated from confined compression test, as shown in the following equation [24]:

(9) $$C_{v}=kE_{c}$$

## 6. Conclusions

We fabricated and characterised a composite silk scaffold by mechanically infusing a matrix component into a porous network scaffold that had a different fibroin concentration. The resulting composite scaffolds performed better in the compression testing with increased elastic modulus and toughness values. This is due to the synergistic effect of network scaffolds elastic response like reinforced fibres and that of the confined matrix hydrogel in the network scaffolds. The composite scaffolds also displayed properties similar to that of cartilage, showing its possible use in such applications. Using foam mechanics, we predicted the mechanical behaviour of the porous network scaffold and the composite scaffold, which helped in understanding the synergistic effects that resulted in enhanced properties. We also determined the fluid transport through the scaffolds under loading, which is an essential characteristic in cartilage like applications. These results are applicable in the development of silk scaffolds for musculoskeletal tissue engineering applications. In addition, the mechanical infusion technique can be used to develop scaffolds with graded mechanical properties by targeted infusion in specific regions. 

## Figures and Tables

**Table 1:** Mechanical properties of network, matrix, and composite scaffolds.

Scaffold	Elastic Modulus (kPa)	Yield Stress (kPa)	Strain at Yielding
**Network**	27.2 ± 9.2	3.8 ± 1.1	0.13 ± 0.03
**Matrix**	140.6 ± 29.8	9.6 ± 1.3	0.12 ± 0.01
**Composite**	478.2 ± 83.9	41.1 ± 3.9	0.16 ± 0.01

**Table 2:** Consolidation coefficients (*C*_v_ mm^2^/min), constrained moduli (*E*_c_ kPa), and hydraulic permeability (*k* m^4^/Ns) are shown for network and composite scaffolds at the different loads.

Parameter	Load (N)	3	6	9	12	15
***C*_v_** **(mm^2^/min)**	**Network**	0.88 ± 0.28	0.38 ± 0.1	0.15 ± 0.03	0.12 ± 0.02	0.07 ± 0.02
	**Composite**	0.65 ± 0.03	0.88 ± 0.04	0.40 ± 0.04	0.30 ± 0.02	0.16 ± 0.01
***E*_c_** **(kPa)**	**Network**	27.7 ± 3.71	46.60 ± 3.95	66.46 ± 4.94	85.06 ± 5.79	104.4 ± 6.83
	**Composite**	72.30 ± 7.99	74.41 ± 6.10	94.02 ± 5.88	110.6 ± 5.86	131.9 ± 6.74
***k ×*** **10^−10^** **(m^4^/Ns)**	**Network**	19.23 ± 5.99	4.90 ± 1.08	1.33 ± 0.20	0.84 ± 0.12	0.39 ± 0.08
	**Composite**	5.47 ± 0.79	7.14 ± 0.61	2.51 ± 0.14	1.64 ± 0.05	0.71 ± 0.03
